# *MCPIP3* as a Potential Metastasis Suppressor Gene in Human Colorectal Cancer

**DOI:** 10.3390/ijms19051350

**Published:** 2018-05-03

**Authors:** Fat-Moon Suk, Chi-Ching Chang, Ren-Jye Lin, Shyr-Yi Lin, Ya-Ting Chen, Yu-Chih Liang

**Affiliations:** 1 Division of Gastroenterology, Department of Internal Medicine, Wan Fang Hospital, Taipei Medical University, Taipei 11696, Taiwan; fmsuk@tmu.edu.tw; 2 Department of Internal Medicine, School of Medicine, College of Medicine, Taipei Medical University, Taipei 11031, Taiwan; ccchang@tmu.edu.tw; 3 Division of Rheumatology, Immunology and Allergy, Taipei Medical University Hospital, Taipei 11031, Taiwan; 4 School of Medical Laboratory Science and Biotechnology, College of Medical Science and Technology, Taipei Medical University, Taipei 11031, Taiwan; linrenjye@tmu.edu.tw (R.-J.L.); a587010603@gmail.com (Y.-T.C.); 5 Department of Primary Care Medicine, Taipei Medical University Hospital, Taipei 11031, Taiwan; sylin@tmu.edu.tw; 6 Department of General Medicine, School of Medicine, College of Medicine, Taipei Medical University, Taipei 11031, Taiwan; 7 Ph.D. Program in Medical Biotechnology, College of Medical Science and Technology, Taipei Medical University, Taipei 11031, Taiwan; 8 Traditional Herbal Medicine Research Center, Taipei Medical University Hospital, Taipei 11031, Taiwan

**Keywords:** MCPIP3, migration, colorectal cancer, epithelial-mesenchymal transition

## Abstract

Monocyte chemotactic protein induced protein 3 (MCPIP3) belongs to the Cys–Cys–Cys–His (CCCH)-zinc finger protein family and contains a highly conserved CCCH-zinc finger domain and a Nedd4-BP1 YacP nuclease (NYN) domain. Previous studies showed that MCPIP3 inhibits the expression of proinflammatory genes, such as vascular cell adhesion molecule (*VCAM*)-1, in human endothelial cells, but the roles and functions of MCPIP3 in cancer cells are still unknown. In human colorectal cancer specimens, we found that the messenger RNA expression of *MCPIP3* was significantly downregulated in cancer tissues compared to adjacent normal tissues (18/25; average fold change of 8.18). Two cell models were used to demonstrate the anti-migration activity of MCPIP3. First, Tet-on T-REx-293/HA-MCPIP3 cells were used to examine whether MCPIP3 can change epithelial–mesenchymal transition (EMT)-related gene expressions. Second, we used two human colorectal cancer cell lines, SW620 and HCT116, to prove the role of MCPIP3 in regulating EMT-related gene expressions. We found that overexpression of MCPIP3 inhibited cell migration according to a wound-healing assay and Transwell invasion assay and vimentin expression, and increased *E*-cadherin expression in these two cell lines. These results suggest that MCPIP3 might play a negative role in cell migration of human colorectal cancer cells.

## 1. Introduction

Colorectal cancer (CRC) ranks third among all cancers in mortality. It is also the third most diagnosed malignant tumor worldwide [1]. The incidence of CRC is related to lifestyle, dietary habits, chronic bacterial infections, and chronic inflammation such as Crohn’s disease. Known risk factors for CRC include smoking, a lack of exercise, obesity, excessive alcohol consumption, and eating red meat or processed meat products [2,3]. CRC metastasis is the primary cause of death in patients. The most common treatment approach for CRC is a surgical operation, and different dose intensities of chemotherapy are administered to patients in accordance with their postoperative pathology reports.

The epithelial–mesenchymal transition (EMT) is a multistage process entailing morphological and phenotypic changes and involves embryonic development and cancer metastasis [4]. While transitioning into mesenchymal cells, epithelial cells lose cell-cell junctions and gain plasticity, mobility, invasive capacity, stem-like characteristics, and an antiapoptotic capacity. During the EMT process, adherent junction proteins, such as the epithelial marker proteins *E*-cadherin, claudin, occluding protein, zonula occludens 1 (ZO-1), and cytokeratins, decrease, thereby reducing cell-cell junctions [5] and enhancing expression of cell adhesion molecules, such as vascular cell adhesion molecule (VCAM)-1, on the surface of epithelial cells of blood vessels. Subsequently, epithelial cells transition into mesenchymal cells and express molecular markers of mesenchymal cells, such as *N*-cadherin, vimentin, and fibronectin, the increases of which induce the EMT process. Many transcription factors can directly or indirectly inhibit *E-cadherin* gene expression. For example, Snail-1, Slug (Snail-2), ZEB1, and ZEB2 can be bound to the promoter of *E-cadherin* to inhibit gene transcription [6].

There are four members in the monocyte chemotactic protein-induced protein (MCPIP) family, namely MCPIP1, MCPIP2, MCPIP3, and MCPIP4, which are Cys–Cys–Cys–His (CCCH)-type zinc finger proteins [7]. MCPIP3 is also called zinc finger CCCH domain-containing protein 12C (ZC3H12C). MCPIP1 plays a vital role in the physiological and pathological processes of inflammation. It effectively inhibits formation of lipopolysaccharide-induced cytokines and the expression of inducible nitric oxide synthase [8]. Studies suggested that MCPIP1 has functions similar to those of ribonuclease (RNase) and can regulate the messenger (m)RNA stability of *interleukin (IL)-6* and *IL-1β* [9,10]; MCPIP1 can also regulate the half-life of *IL-1β* mRNA. Overexpression of MCPIP1 inhibits the binding between nuclear factor (NF)-κB and DNA, thereby reducing NF-κB activity [8,11]. Previous studies identified a mutation of the *MCPIP4* gene in several types of carcinoma tissue. Therefore, *MCPIP4* was inferred to be a potential tumor-suppressing gene [12,13,14], which inhibits cell growth, exhibits RNase activity, and regulates specific RNA contents. Recent studies identified polyubiquitination as being critical in promoting proliferation and cell survival signaling pathways such as those for protein kinase B (Akt) and NF-κB cells [15,16]. Studies found that MCPIP1 can facilitate the deubiquitination of tumour necrosis factor receptor-associated factor 6 (TRAF6) ligase by an ubiquitin specific peptidase 10 (USP10)-dependent manner [17,18] and MCPIP4 decreases the global cellular protein ubiquitination [19] However, the functions and molecular mechanisms of MCPIP2 require further research.

The protein structure of MCPIP3 resembles that of MCPIP1, consisting of a highly conserved CCCH-zinc finger domain situated in the center of the protein and a Nedd4-BP1 YacP nuclease (NYN) domain in front of the CCCH-zinc finger domain [20]. Although MCPIP3 and MCPIP1 share similar amino acid sequence structures, few studies have been conducted on MCPIP3. Previous research revealed that MCPIP3 inhibits tumor necrosis factor (TNF)-α-induced NF-κB activity and negatively regulates inflammation of endothelial cells of blood vessels and the expression of cell adhesion molecules such as IL-8, monocyte chemoattractant protein (MCP)-1, VCAM-1, ICAM-1, and *E*-selectin [20]. However, the molecular mechanism of MCPIP3 in inhibiting NF-κB activity remains unclear.

In this study, we collected 25 pairs of T3 CRC tissues and adjacent normal tissues. The results revealed that *MCPIP3* mRNA expression in tumor tissues was lower. Further research showed that overexpression of MCPIP3 changed the expression of EMT-related genes. Therefore, MCPIP3 appears to play a central role in tumor metastasis.

## 2. Results

### 2.1. Downregulation of MCPIP3 in Human CRC Tissues

**Figure 1:**
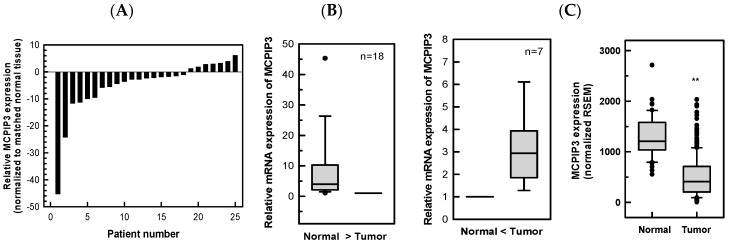
*MCPIP3* mRNA levels of paired human colorectal cancer (CRC) tissues and adjacent normal tissues. The mRNA expression levels of *MCPIP3* from 25 paired human CRC tissues and adjacent normal tissues were determined by a real-time RT-PCR. (**A**) Expression levels are shown as the change vs. the matching normal adjacent tissue. (**B**) Of these, 18 tissue pairs had a higher expression of *MCPIP3* in normal tissues than tumor tissues, while seven pairs had opposite results. (**C**) The clinical data and mRNAseq data of colorectal adenocarcinoma (COADREAD) were from Firebrowse (http://firebrowse.org/) data portal with TCGA data version 2016_01_28. Box plots of *MCPIP3* expression in colorectal cancer using independent tumor (*n* = 373) and normal (*n* = 51) data. RSEM, RNA-seq by expectation maximization. ** *p* < 0.01.

In total, 25 pairs of T3 CRC tissues and adjacent normal tissues were collected from patients with CRC. After total RNA was extracted, real-time reverse transcription-polymerase chain reaction (RT-PCR) was employed to analyze the expression of *MCPIP3* mRNA. As shown in Figure 1A, the relative level of *MCPIP3* mRNA in 18/25 (72.0%) tumor tissues showed a decrease in the *MCPIP3* mRNA level, compared with that of the adjacent normal tissues; specifically, the average expression in adjacent normal tissues was approximately 8.18-fold that in tumor tissues. In the remaining seven patients, *MCPIP3* mRNA expression in tumor tissues was higher than that in peripheral normal tissues, rendering an average expression in tumor tissues 3.17-fold that in adjacent normal tissues. Data obtained from The Cancer Genome Atlas (TCGA) were also analyzed and found that levels of *MCPIP3* mRNA were significantly decreased in majority of CRC samples as compared with normal tissue samples (Figure 1C). The results suggest that *MCPIP3* expression in CRC tumor tissues is lower than that in normal tissues. Thus, MCPIP3 may play a role in inhibiting tumor growth or metastasis.

In addition, we also analyzed the relationships between *MCPIP3* mRNA expression level and patient survival as well as *MCPIP3* mRNA expression level and tumor stage of human CRC using the database of The Human Protein Atlas (https://www.proteinatlas.org/). Interestingly, high levels of *MCPIP3* mRNA were correlated with high survival rate of CRC patients (Figure S1A). The more malignant tumors tended to express lower amounts of *MCPIP3* at the mRNA level. However, the significant difference was found only between stage I and stage III as well as stage I and stage IV (Figure S1B). The results suggest that *MCPIP3* expression in CRC tumor tissues is lower than that in normal tissues and can be used to predict patient survival and might be involved in inhibition of tumor migration and invasion. Thus, MCPIP3 may play a role in inhibiting tumor growth or metastasis.

### 2.2. Overexpression of MCPIP3 Inhibits Cell Migration in T-REx-293/HA-MCPIP3 Cells

To examine the role of MCPIP3 in cells, a T-REx-293/HA-MCPIP3 cell line was established by transducing an *HA-MCPIP3* gene into Tet-on T-REx-293 cells. HA-tagged MCPIP3 is expressed under the presence of the tetracycline derivative doxycycline (Dox). To determine whether MCPIP3 regulates cell growth, 1 μg/mL Dox was employed to treat T-REx-293/HA-MCPIP3 cells for 1, 2, 4, and 5 days. A 3-(4,5-dimethyl-2-thiazolyl)-2,5-diphenyl-2-H-tetrazolium bromide (MTT) assay was used to measure viable cells, revealing that the viability of Dox-treated cells was slightly lower than that of control cells; however, the difference was nonsignificant (Figure S2). The experimental results suggest that in T-REx-293/HA-MCPIP3 cells, overexpression of MCPIP3 did not influence cell survival.

**Figure 2:**
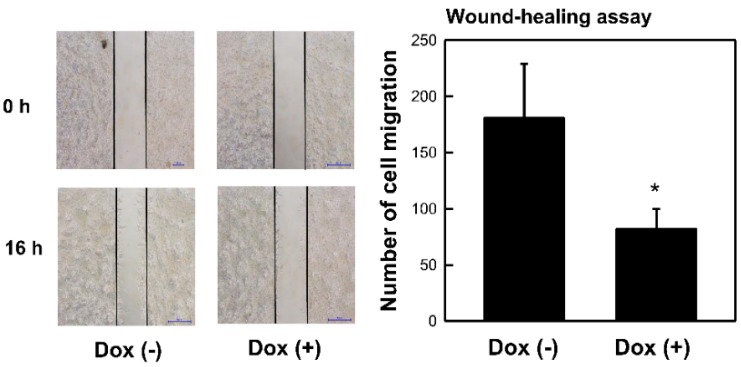
Effects of MCPIP3 overexpression on cell migration of T-REx 293/HA-MCPIP3 cells. Cells were treated with 1 μg/mL of doxycycline (Dox) for 16 h, and the number of cells migrating was determined by a wound-healing assay (magnification ×200). A representative photo and quantified data are shown. Each value represents the mean ± SE from three independent experiments. * *p* < 0.05 vs. doxycycline-free cells.

A wound-healing assay was used to understand whether MCPIP3 expression affects cell migration. T-REx-293/HA-MCPIP3 cells were treated with Dox (1 μg/mL) for 16 h. A microscope was used to observe cell migration and count the amount of cell migration. Results showed that overexpression of MCPIP3 inhibited cell migration (Figure 2), verifying the capability of the protein to downregulate cell migration.

### 2.3. Overexpression of MCPIP3 Affects EMT-Related Protein Expressions in T-REx-293/HA-MCPIP3 Cells

**Figure 3:**
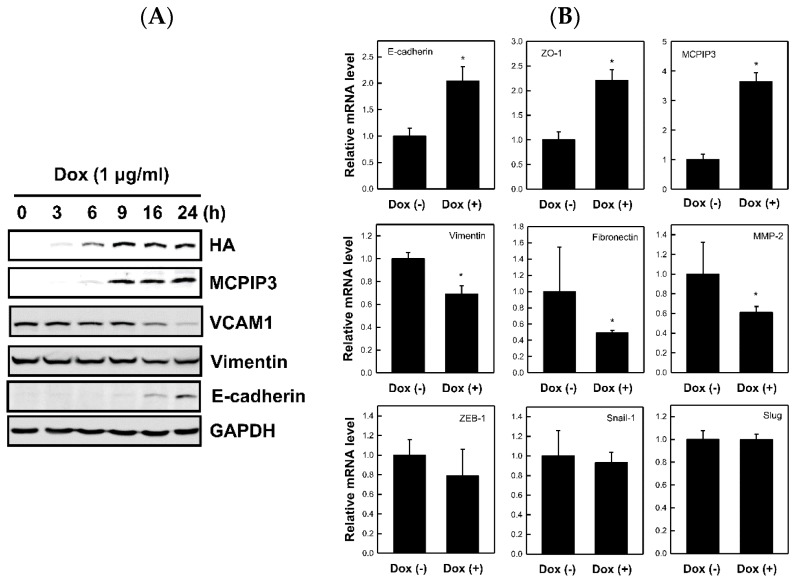
Time-dependent effects of MCPIP3 overexpression on protein expressions of epithelial–mesenchymal transition (EMT)-related marker genes in T-REx 293/HA-MCPIP3 cells. (**A**) Cells were treated with 1 μg/mL of doxycycline (Dox) for 3, 6, 9, 16, and 24 h. Total proteins were collected to determining protein expressions of EMT-related marker genes by a Western blot analysis. (**B**) Cells were treated with 1 μg/mL of doxycycline for 9 h. Total RNA was collected to determine mRNA expressions of EMT-related marker genes by a real-time RT-PCR analysis. Each value represents the mean ± SE from three independent experiments. * *p* < 0.05 vs. doxycycline-free cells. VCAM1: vascular cell adhesion molecule 1; GAPDH: glyceraldehyde-3-phosphate dehydrogenase.

The EMT process is vital to tumor cell migration. To determine whether MCPIP3 changes EMT-related molecule expressions to influence cell migration, we overexpressed MCPIP3 in T-REx-293/HA-MCPIP3 cells and analyzed expressions of crucial EMT-related molecules, such as *E*-cadherin (an epithelial marker protein) and vimentin (a mesenchymal marker protein). Figure 3A shows that expressions of the MCPIP3 and *E*-cadherin proteins increased with a prolonged Dox treatment duration. In contrast, expressions of the vimentin and VCAM-1 proteins declined with a prolonged Dox treatment duration. In a test of whether overexpression of MCPIP3 influences *E-cadherin* and *vimentin* mRNA expressions, as shown in Figure 3B, Dox-treated cells demonstrated increased *E-cadherin* mRNA expression and decreased *vimentin* mRNA expression. In addition, overexpression of MCPIP3 enhanced mRNA expressions of other epithelial markers, such as *ZO-1*, and repressed mRNA expressions of other mesenchymal markers, such as *fibronectin*. The mRNA expressions of other EMT-related transcription factors, such as *ZEB1*, *Snail1*, and *Slug*, were not affected by the overexpression of MCPIP3. Results suggest that MCPIP3 can inhibit cell migration by changing EMT-related gene expressions.

### 2.4. Overexpression of MCPIP3 Inhibits Cell Migration and Affects EMT-Related Gene Expressions in Human CRC Cells

**Figure 4:**
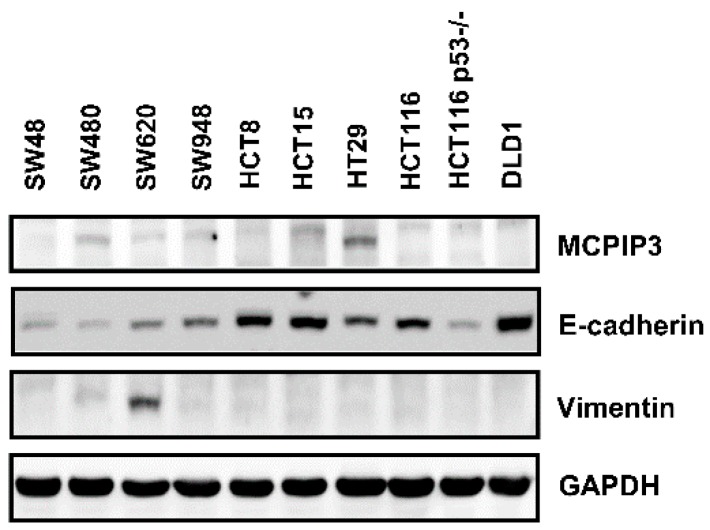
Protein expression levels of MCPIP3 in the ten human colorectal cancer cell lines. SW48, SW480, SW620, SW948, HCT8, HCT15, HT29, HCT116 wild-type, HCT116 p53^−/−^, and DLD1 cells were cultured for 24 h, and then total proteins were collected to determine MCPIP3 protein expression by a Western blot analysis.

To further confirm whether MCPIP3 regulates cancer cell migration and changes EMT-related molecule expressions in human CRC cells, we selected 10 human CRC cell lines, namely SW48, SW480, SW620, SW948, HCT8, HCT15, HT29, HCT116 p53 wild-type, HCT116 p53^−/−^, and DLD1. *E*-Cadherin and vimentin protein expressions of these cells lines were tested, revealing that in all human CRC cell lines, MCPIP3 protein expression was low (Figure 4), among which the HT29 strain exhibited relatively higher expression of MCPIP3. Next, the SW620 and HCT116 cell lines were selected for subsequent research because SW620 has properties of mesenchymal cells, expressing high vimentin and low *E*-cadherin; in contrast, HCT116 exhibits epithelial cell characteristics, expressing high *E*-cadherin and low vimentin.

**Figure 5:**
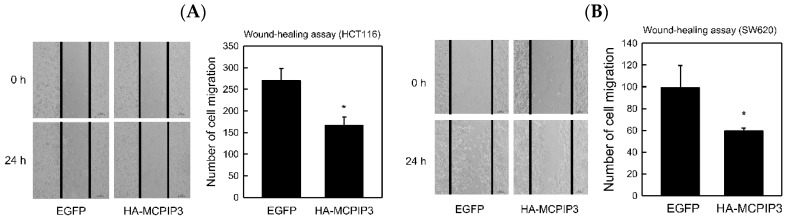
Effects of MCPIP3 overexpression on the cell migration of human HCT116 and SW620 colorectal cancer cells. (**A**) HCT116 and (**B**) SW620 cells were transduced with lentiviral vectors (at an MOI of 3) expressing the indicated enhanced green fluorescent protein (EGFP; as a control) and HA-MCPIP3 for 48 h, and the cell migration of equal amounts of transduced cells was examined by a wound-healing assay (magnification ×200) for 24 h. Each value represents the mean ± SE from three independent experiments. * *p* < 0.05 vs. EGFP control cells.

These two cell lines were transduced with a lentivirus (at a multiplicity of infection (MOI) of 3) carrying the pSIN/HA-MCPIP3 plasmid, and in control experiments, cells were transduced with a lentivirus carrying the pSIN/EGFP plasmid. After SW620 and HCT116 cells were transduced with the lentivirus for 1–3 days, an MTT assay was applied to analyze cell survival. Results revealed that overexpression of MCPIP3 slightly decreased cell survival, but this decline did not reach statistical significance (Figure S3). In T-REx-293/HA-MCPIP3 cells, overexpression of MCPIP3 also demonstrated a similar trend (Figure S2). If cells are cultivated with an increased MOI or prolonged treatment time, we cannot rule out the possibility that overexpression of MCPIP3 can inhibit cell growth and eventually cause cell apoptosis. Since we have found that overexpression of MCPIP3 for 5 days resulted in significant decrease of cell proliferation (Figure S4). The mechanism for this, however, will require further clarification from a future in-depth study. Western blotting was adopted to determine that the lentivirus facilitated successful expression of MCPIP3 in the two cell lines (Figure S3B). Subsequently, the lentiviral transduction mode was used to test whether overexpression of MCPIP3 inhibited human CRC cell migration and invasion. For both SW620 and HCT116 cells, the wound-healing assay showed that overexpression of MCPIP3 significantly inhibited cell migration (Figure 5).

**Figure 6:**
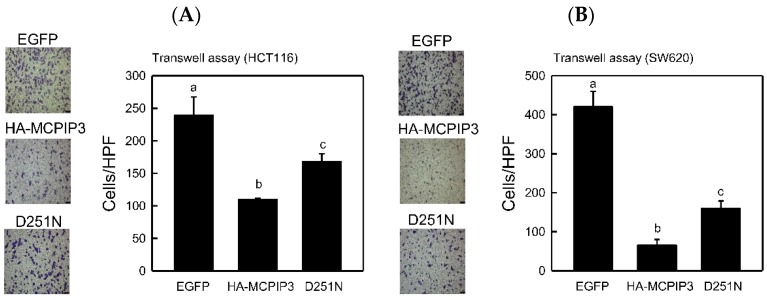
Effects of MCPIP3 and MCPIP3-D251N mutant overexpression on the cell invasiveness of human HCT116 and SW620 colorectal cancer cells. (**A**) HCT116 and (**B**) SW620 cells were transduced with a lentiviral vector (at an MOI of 3) expressing the indicated enhanced green fluorescent protein (EGFP), HA-MCPIP3, and HA-MCPIP3-D251N mutant for 48 h, and cell invasion by equal amounts of transduced cells was examined with a Transwell invasion assay for 24 h. Migrated cells were stained with crystal violet and cell numbers were counted in a high-power field (HPF) (magnification ×200). Each value represents the mean ± SE from three independent experiments. Values not sharing a same superscript letter are significantly different (*p* < 0.05).

**Figure 7:**
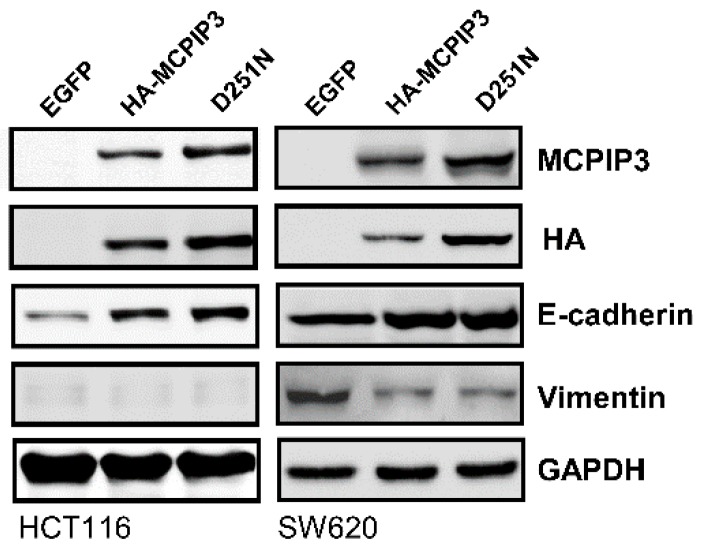
Effects of MCPIP3 and MCPIP3-D251N mutant overexpression on epithelial–mesenchymal transition (EMT)-related protein expressions in human HCT116 and SW620 colorectal cancer cells. HCT116 and SW620 cells were transduced with a lentiviral vector (at an MOI of 3) expressing the indicated enhanced green fluorescent protein (EGFP), HA-MCPIP3, and HA-MCPIP3-D251N mutant and then cultured for 24 h. Total proteins were collected to determine protein expressions by a Western blot analysis.

The NYN domain of MCPIP3 has RNase activity, but the D251N mutation in the NYN domain abolishes its RNase activity. To examine whether RNase activity of the NYN domain involves inhibiting cell invasion, we used a lentiviral expressing the HA-tagged MCPIP3 or MCPIP3-D251N mutant. As shown in Figure 6, the Transwell invasion assay revealed that both MCPIP3 and MCPIP3-D251N significantly inhibited cell invasion and loss of RNase activity decreased the inhibitory activity of MCPIP3 on cell invasion. The results suggest that the RNase activity of MCPIP3 plays a minor role in inhibition of cell invasion. Moreover, the overexpression of MCPIP3 and the MCPIP3-D251N mutant enhanced *E*-cadherin expression and inhibited vimentin expression as in T-REx-293/HA-MCPIP3 cells (Figure 7). However, there was no significant difference in change of *E*-cadherin and vimentin expression between MCPIP3-overexpressed and MCPIP3-D251N-overexpressed cells. These results verified that in human CRC cells, MCPIP3 can change EMT-related gene expressions to inhibit cancer cell migration and invasion, and the RNase activity of MCPIP3 might be not involved in the regulation of *E*-cadherin and vimentin expression. In addition, the *E*-cadherin can bind with β-catenin and form complexes, and the aberrant expression of *E*-cadherin/β-catenin complexes is correlated with the EMT processes [21]. Overexpression of MCPIP3 did not change the expression of β-catenin (Figure S5), suggesting β-catenin is not involved in the EMT processes regulated by MCPIP3.

## 3. Discussion

This study analyzed 25 pairs of human CRC tissue specimens. According to the TNM cancer staging system (T means “the extent of the tumor”; N means “the extent of spread to the lymph nodes”; M means “the presence of metastasis”), these carcinoma tissues were T3 primary tumors, which entails tumor invasion penetrating the muscularis propria to the mucosa. A real-time RT-PCR was applied to analyze tumor tissues and adjacent normal tissues for *MCPIP3* mRNA expression, revealing that most tumor tissues expressed a lower level of MCPIP3 than adjacent normal tissues. Thus, MCPIP3 possibly plays a crucial role in CRC cell growth and/or metastasis. In T-REx-293/HA-MCPIP3, SW620, and HCT116 cells, overexpression of MCPIP3 did not influence cell proliferation but did inhibit cell migration and invasion. Furthermore, overexpression of MCPIP3 changed EMT-related gene expressions, including increasing *E-cadherin* expression and decreasing *vimentin* expression. Therefore, the results suggest that MCPIP3 plays a crucial role in downregulating human CRC cell migration.

MCPIP family members contain both a CCCH zinc finger domain (relevant to DNA/RNA binding) and an NYN nuclease domain (which has RNase activity). Previous studies focused on MCPIP1 and MCPIP4. The main function of MCPIP1 is as an antiviral, and its feedback downregulates inflammation. MCPIP4 was speculated to be a tumor-suppressor gene, because its mutation was identified in certain tumors. Previous studies indicated that the CCCH zinc finger domain of MCPIP1 can bind to AU-rich elements (AREs) of mRNA (e.g., *TNF-α* and *GM-CSF*), thereby causing these mRNAs to become unstable and degrade [10]. However, it was demonstrated that MCPIP1 can degrade the proinflammatory molecule, *IL-1β*, through an ARE-independent pathway [22]. The current study demonstrated that MCPIP3 can enhance *E-cadherin* mRNA expression and inhibit *vimentin* mRNA expression, and the underlying molecular mechanism seems to be different between regulation of *E-cadherin* and *vimentin* by MCPIP3.

Regarding to the downregulation of *vimentin* by MCPIP3, we noted that the AREs are not present within the 3′UTR of *vimentin* mRNA [23]. In addition, the MCPIP3-D251N mutant did not lose its inhibitory activity on the vimentin protein expression (Figure 7). Therefore, we think the downregulation of vimentin expression by MCPIP3 might not be associated with the RNase activity of MCPIP3. It has been known that activator protein 1 (AP-1) consensus element is a stronger enhancer located in human *vimentin* gene promoter, and 12-O-tetradecanoyl-phorbol-13-acetate (TPA) and growth factors can induce *vimentin* gene expression by activation of AP-1 [24,25]. Previous study also found that MCPIP1 functions as a negative regulator of AP-1 signaling pathways. It is possible that MCPIP3 negatively regulates AP-1 activity as MCPIP1 does, and then down-regulates vimentin expression [26].

On the other hand, the upregulation of *E-cadherin* by MCPIP3 might be at the transcription level. Many transcription factors that can directly or indirectly inhibit *E-cadherin* are regarded as EMT-related transcription factors, such as Snail1, Slug (Snail2), ZEB1, and ZEB2. These transcription factors can bind to the promoter of *E-cadherin* and inhibit *E-cadherin* gene expression [6]. However, results of the present study show that overexpression of MCPIP3 did not influence mRNA expressions of *Snail1*, *Slug* (*Snail2*), or *ZEB1* (Figure 3B); thus, we think that MCPIP3 does not change *E-cadherin* expression by changing expressions of these transcription factors. Nonetheless, we cannot exclude the possibility that MCPIP3 may change the transcription activities of these factors, or directly or indirectly binds to a gene promoter and then influence EMT-related gene expressions. Moreover, matrix metalloproteinases (MMPs) of basement membranes, of which MMP-2 and MMP-9 are considered the most essential in cell metastasis [27], can hydrolyze collagen and the extracellular matrix. The present study found that overexpression of MCPIP3 inhibited *MMP-2* mRNA expression (Figure 3B), indicating that MCPIP3 may inhibit *MMP* gene expression and thus tumor cell metastasis. Previous studies indicated that VCAM-1 expression influences EMT-related gene expressions [28]. High expression of VCAM-1 reduces *E*-cadherin expression and elevates vimentin expression, thereby enhancing cell adhesion, migration, and invasion. Both a previous study [20] and the current study identified that overexpression of MCPIP3 can effectively inhibit VCAM-1 expression (Figure 3). Accordingly, overexpression of MCPIP3 can be mediated through down-regulation of VCAM-1 molecules, which in turn helps maintains epithelial phenotype, thereby weakening cell migration and invasion.

Analyzing CRC tissues of clinical patients showed that mRNA expression of *MCPIP3* in most CRC tissues was low (Figure 1), but it increased in a small number of CRC tissues. Previous studies indicated that the MCPIP family can negatively regulate macrophage activation [7,8]. Macrophages in later-period inflammation induce *MCPIP* gene expression, which forms a further feedback loop to inhibit macrophage inflammation. Tumor tissues generally demonstrate infiltration of inflammatory macrophages; thus, the inflammation status involving macrophages may influence MCPIP expression. In this study, a great number of macrophages may have existed in some of tumor tissues, and therefore MCPIP3 expression may have been influenced by these macrophages.

## 4. Materials and Methods

### 4.1. Cell Lines, Antibodies, and Patient Samples

Human embryonic kidney HEK293 cells and tetracycline (Tet)-regulated expression embryonic kidney T-REx-293/HA-MCPIP3 cell lines were kindly provided by Yi-Ling Lin (Institute of Biomedical Sciences, Academia Sinica, Taipei, Taiwan) and grown in Dulbecco’s modified Eagle’s medium (DMEM) containing 10% fetal bovine serum (FBS). Human colon adenocarcinoma SW48, SW480, SW948, HCT8, HCT15, HCT116, HT29, and DLD-1 cells were obtained from the Food Industry Research and Development Institute (FIRDI, Hsinchu, Taiwan) and grown according to FIRDI suggestions. Rabbit polyclonal anti-MCPIP3 (OriGene Technologies, Rockville, MD, USA), mouse monoclonal anti-heme agglutinin (HA) (Covance, Princeton, NJ, USA), rabbit polyclonal anti-GAPDH (GeneTex, Irvine, CA, USA), rabbit polyclonal anti-*E*-cadherin (Cell Signaling Technology, Danvers, MA, USA), rabbit polyclonal anti-vimentin (GeneTex), rabbit polyclonal anti-snail1 (GeneTex), and rabbit polyclonal anti-β-catenin (GeneTex) were used in this study.

Human CRC tissues and paired adjacent normal tissues (*n* = 25) were obtained as anonymous surgical specimens from Taipei Medical University Hospital (TMUH), and TMUH Institutional Review Board (IRB) approved the study with a waiver of informed consent (protocol no. CRC-12-11-06; approval date, 29 June, 2011). All cancer tissues came from patients with T3 stage CRC according to the tumor-node-metastasis (TNM) staging system of the American Joint Committee on Cancer (AJCC).

### 4.2. Plasmids and Lentivirus

Complementary DNA (cDNA) of human *MCPIP3* with an N-terminal HA-tag was subcloned into a lentiviral expression construct (pSIN), named pSIN-HA-MCPIP3. The pSIN HA-MCPIP1-D251N was generated from a pSIN-HA-MCPIP3 wild-type construct. The lentiviral vector plasmid, pSIN-GFP, containing an *enhanced green fluorescent protein* (*EGFP*) gene served as the control plasmid. These three plasmids were kindly provided by Yi-Ling Lin, and the generation and titer of lentiviruses were based on a previously described method [29].

### 4.3. Western Blotting

Total cellular proteins (30–50 μg) were prepared and then fractionated by sodium dodecylsulfate polyacrylamide gel electrophoresis (SDS-PAGE) as described previously [30]. After being transferred to a polyvinylidene difluoride (PVDF) membrane, the membrane was blocked in 1% bovine serum albumin (BSA) for 1 h before being incubated with specific primary antibodies. The secondary antibodies conjugated to horseradish peroxidase (HRP) were used to detect antigen–antibody complexes using an enhanced chemiluminescence (ECL, Thermo Fisher Scientific Taiwan, Taipei, Taiwan) kit in an ImageQuant LAS 4000 Biomolecular Imager (GE Healthcare Life Sciences, Marlborough, MA, USA).

### 4.4. Real-Time Reverse Transcription-Polymerase Chain Reaction (RT-PCR)

Total RNA was isolated with a Trizol RNA extraction reagent [31] and cDNA was synthesized using a GoScript™ Reverse Transcription System (Promega, Madison, WI, USA) according to the manufacturer’s instructions. One microgram of cDNA was used to perform the real-time PCR on an Applied Biosystems StepOne Real-Time PCR System (Applied Biosystems, Foster City, CA, USA) with Applied Biosystems™ SYBR™ Green PCR Master Mix (Applied Biosystems, Foster City, CA, USA) according to the manufacturer’s instructions. A total volume of 20 μL contained 1 μg cDNA, 0.2 μM of each primer, and 10 μL SYBR™ Green PCR Master Mix. Primer sequences are shown in Supplementary Table S1. The conditions of the real-time PCR were: 95 °C pre-incubation for 20 s; 40 cycles of 95 °C for 20 s, 60 °C for 30 s, and 72 °C for 30 s for amplification; and 95 °C for 15 s, 60 °C for 1 min, and 95 °C for 15 s for a melting curve analysis. Expression levels of each mRNA were calculated using the comparative *C*t method (2d*C*t formula) and were normalized to the GAPDH mRNA level [32].

### 4.5. Cell Viability Assay

At the end of the experiments, cells were washed with phosphate-buffered saline (PBS) twice, and incubated with 200 μL of a 3-(4,5-dimethyl-2-thiazolyl)-2,5 diphenyl-2*H*-tetrazolium, thiazolyl blue tetrazolium bromide (MTT, Sigma Chemical, St. Louis, MO, USA) solution at 37 °C for 30 min. After removing the MTT solution, 200 μL dimethyl sulfoxide (DMSO) was added to each cell, and then the absorbance at 540 nm was measured with an enzyme-linked immunosorbent assay (ELISA) reader [33].

### 4.6. Wound-Healing Assay

Cells were seeded in reservoirs of a Culture-Insert (ibidi GmbH, Martinsried, Germany) which was stuck to the surface of a 12-well cell culture plate. The Culture-Insert was then removed, and cells were continuously cultured for 24 h. Four low-magnification areas (100×) were randomly captured by an inverted microscope (Nikon ECLIPSE TE2000-U, Kanagawa, Japan), and the number of migrated cells was visually counted [34].

### 4.7. Invasion Assay

Cells were seeded in the upper chamber of a Transwell (Corning Costar, Corning, NY, USA), which was coated with Matrigel^®^ matrix (Corning Life Sciences, Taipei, Taiwan), and medium with 10% fetal bovine serum (FBS) was added to the lower chamber. After 24 h, cells in the top of the filter were removed with a cotton swab, and cells in the bottom of the filter were fixed with 34% paraformaldehyde and stained with 0.1% crystal violet dye. Four low-magnification areas (100×) were randomly captured with an inverted microscope (Nikon ECLIPSE TE2000-U), and the number of invaded cells was counted [34].

### 4.8. Statistical Analysis

Statistical analyses were performed using one-way Student’s *t*-test by SigmaPlot 13.0 (Systat Software Inc., San Jose, CA, USA), and differences were considered significant at *p* < 0.05.

## Supplementary Materials

Supplementary materials can be found at http://www.mdpi.com/1422-0067/19/5/1350/s1.
