# Supplementary material for: MCPIP3 as a Potential Metastasis Suppressor Gene in Human Colorectal Cancer
Source: Int J Mol Sci. 2018 May 3;19(5):1350. doi: 10.3390/ijms19051350 (PMC5983627; doi:10.3390/ijms19051350)
Supplement: Supplementary file 1 [file ijms-19-01350-s001.pdf]

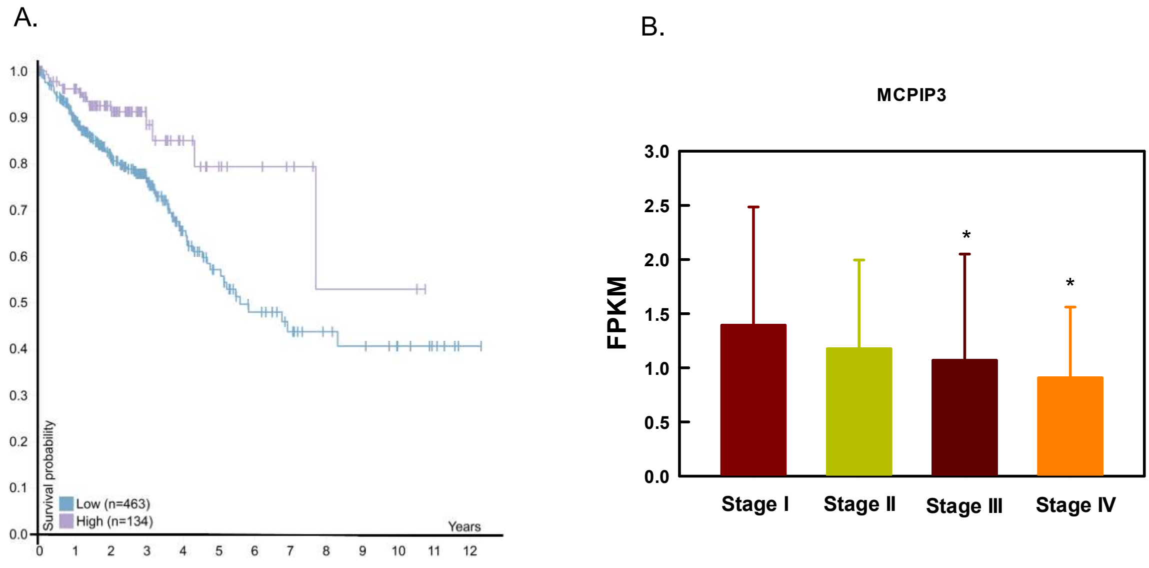

Figure S1. The relationships between *MCPIP3* mRNA expression level and patient survival as well as *MCPIP3* mRNA expression level and tumor stage of human CRC using TCGA database. (A) The Kaplan-Meier plot and (B) the tumor stages data were obtained from The Human Protein Atlas (https://www.proteinatlas.org/ENSG00000149289-ZC3H12C/pathology/tissue/colorectal+cancer#Intensity), and (B) each value represents the mean±SD by one-way ANOVA analysis. FPKM, fragments per kilobase of exon model per million reads mapped. **P* <0.05 vs. stage I.

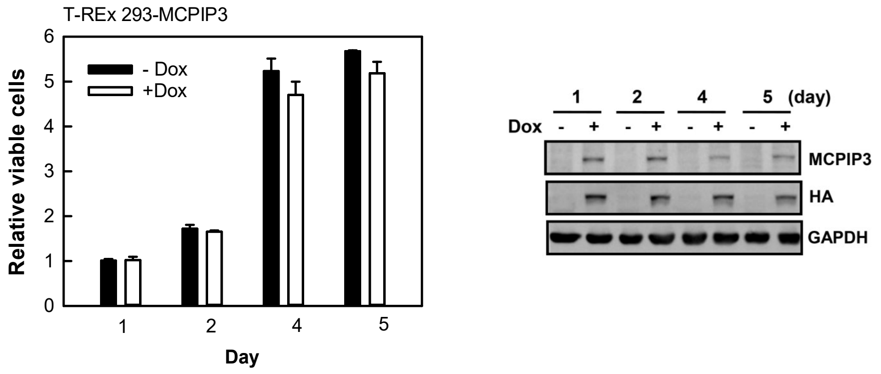

Figure S2. Effects of MCPIP3 overexpression on the cell viability of T-REx 293/HA-MCPIP3 cells. HA-MCPIP3 was induced by 1 μg/ml of doxycycline (Dox) for 1~5 days, viable cells were determined by an MTT method, and the HA-MCPIP3 protein expression were detected by Western blot. Each value represents the mean ± SE from three independent experiments.

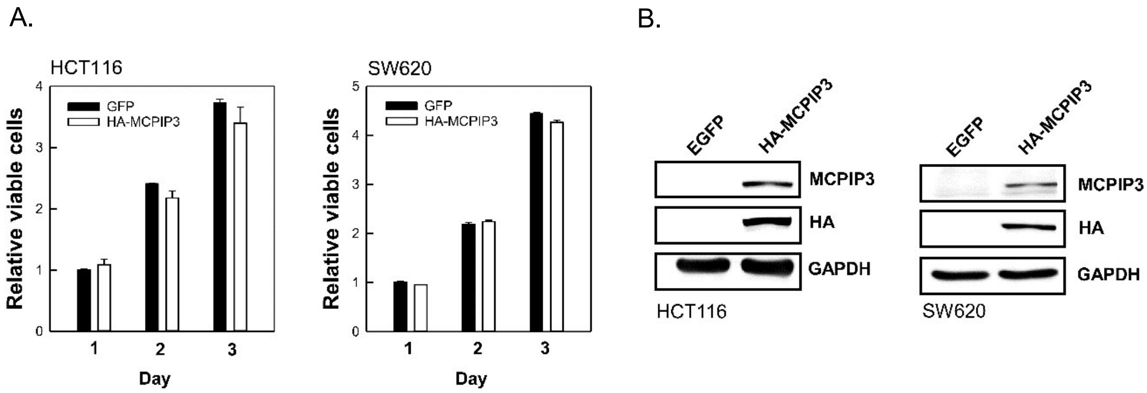

Figure S3. Effects of MCPIP3 overexpression on the cell viability in human HCT116 and SW620 colorectal cancer cells. (A) Cells were transduced with lentiviral vectors (at an MOI of 3) expressing the indicated enhanced green fluorescent protein (EGFP; as a control) and HA-MCPIP3 for 1~3 days, and viable cells were determined with an MTT analysis. Each value represents the mean ± SE from three independent experiments. (B) The expression of HA-MCPIP3 was confirmed by determining the protein expression using a Western blot analysis.

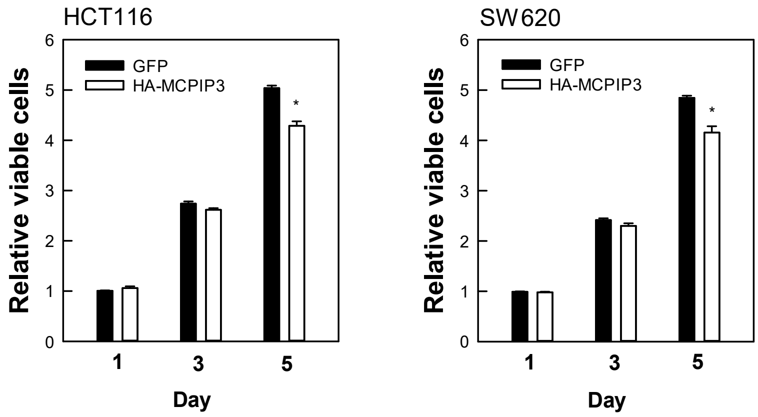

Figure S4. Effects of MCPIP3 overexpression on the cell viability in human HCT116 and SW620 colorectal cancer. (A) Cells were transduced with lentiviral vectors (at an MOI of 3) expressing the indicated enhanced green fluorescent protein (EGFP; as a control) and HA-MCPIP3 for 1~5 days, and viable cells were determined with an MTT analysis. Each value represents the mean ± SE from three independent experiments. **P*<0.05.

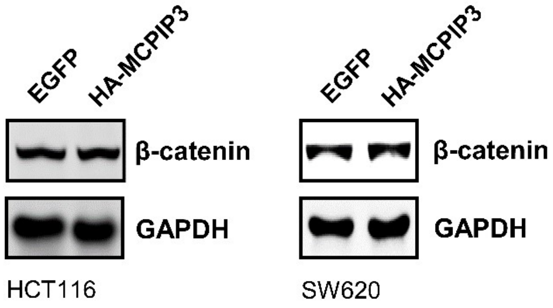

Figure S5. Effects of MCPIP3 overexpression on the β-catenin protein expressions in human HCT116 and SW620 colorectal cancer cells. HCT116 and SW620 cells were transduced with a lentiviral vector (at an MOI of 3) expressing the indicated enhanced green fluorescent protein (EGFP) and HA-MCPIP3 and then cultured for 24 h. Total proteins were collected to determine protein expressions by a Western blot analysis.

Supplementary Table S1. The primer sequences used in this study.

Name		Sequences
E-cadherin	Forward	5′-TTCTGGAAGGAATGGAGGAGTC-3′
	Reverse	5′-ACCTGGAATTGGGCAAA TGTG-3′
Fibronetin	Forward	5′-CCCACCGTCTCAACATGCTTAG-3′
	Reverse	5′-CTCGGCTTCCTCCATAACAAGTAC-3′
GAPDH	Forward	5′-AGCCACATCGCTCAGACAC-3′
	Reverse	5′-GCCCAATACGACCAAATCC-3′
MCPIP3	Forward	5′-AACCAATCCACTGCCAATTT-3′
	Reverse	5′-TTGATGGCAGCAATGTGG-3′
MMP2	Forward	5′-CAAC TACGATGATGACCGCAA-3′
	Reverse	5′-GTGTAAAT GGGTGCCATCAGG3′
Slug	Forward	5′-AGATGCATATTCGGACCCAC-3′
	Reverse	5′-CCTCATGTTTGTGCAGGAGA-3′
Vimentin	Forward	5′-AGATGGCCCTTGACATTG AG-3′
	Reverse	5′-TGGA AGAGGCAGAGAAATCC-3′
ZEB-1	Forward	5′-GCACAACCAAGTGCAGAAGA-3′
	Reverse	5′-GCCTGGTTCAGGAGAAG ATG-3′
ZO-1	Forward	5′-GTGTTGTGGATACCTTGT-3′
	Reverse	5′-GATGATGCCTCGTTCTAC-3′
